# The Prevalence and Use of Walking Loops in Neighborhood Parks: A National Study

**DOI:** 10.1289/EHP293

**Published:** 2016-08-12

**Authors:** Deborah A. Cohen, Bing Han, Kelly R. Evenson, Catherine Nagel, Thomas L. McKenzie, Terry Marsh, Stephanie Williamson, Peter Harnik

**Affiliations:** 1 RAND Corporation, Santa Monica, California, USA; 2 Department of Epidemiology, UNC Gillings School of Global Public Health, Chapel Hill, North Carolina, USA; 3 City Parks Alliance, Washington, DC, USA; 4 School of Exercise and Nutritional Sciences, San Diego State University, San Diego, California, USA; 5 Trust for Public Land, Washington, DC, USA

## Abstract

**Background:**

Previous studies indicate that the design of streets and sidewalks can influence physical activity among residents. Park features also influence park use and park-based physical activity. Although individuals can walk on streets and sidewalks, walking loops in parks offer a setting to walk in nature and to avoid interruptions from traffic.

**Objectives:**

Here we describe the use of walking loops in parks and compare the number of park users and their physical activity in urban neighborhood parks with and without walking loops.

**Methods:**

We analyzed data from the National Study of Neighborhood Parks in which a representative sample of neighborhood parks (n = 174) from 25 U.S. cities with > 100,000 population were observed systematically to document facilities and park users by age group and sex. We compared the number of people and their physical activity in parks with and without walking loops, controlling for multiple factors, including park size, facilities, and population density.

**Results:**

Overall, compared with parks without walking loops, on average during an hourly observation, parks with walking loops had 80% more users (95% CI: 42, 139%), and levels of moderate-to-vigorous physical activity were 90% higher (95% CI: 49, 145%). The additional park use and park-based physical activity occurred not only on the walking loops but throughout the park.

**Conclusions:**

Walking loops may be a promising means of increasing population level physical activity. Further studies are needed to confirm a causal relationship.

**Citation:**

Cohen DA, Han B, Evenson KR, Nagel C, McKenzie TL, Marsh T, Williamson S, Harnik P. 2017. The prevalence and use of walking loops in neighborhood parks: a national study. Environ Health Perspect 125:170–174; http://dx.doi.org/10.1289/EHP293

## Introduction

The importance of increasing physical activity and preventing chronic disease is highlighted in the recent Step it Up! Campaign, which is the *Surgeon General’s Call to Action to Promote Walking and Walkable Communities* (DHHS 2015). This call to action not only encourages individuals to increase their physical activity, but also urges local jurisdictions to design communities to be more pedestrian friendly.

Neighborhood parks are usually between 2 and 20 acres and are intended to serve local residents living within a 1-mi radius (Mertes and Hall 1996). They provide an infrastructure that allows residents of all ages to recreate there on a routine basis. Because they typically contain diverse facilities for play, sport, and exercise, neighborhood parks are a community resource that supports population physical activity.

Some neighborhood parks have relatively long (usually ≥ 0.5 mi), uninterrupted pathways specifically designed for walking, biking, or other nonmotorist recreational activity that typically preclude intrusions from other uses. These paths are often loops that have a circular design, but are occasionally curvilinear where the beginning and end of the path do not meet. These paths can themselves be a destination for park users and they are distinct from park sidewalks, which are usually shorter and designed mainly to connect park destinations (e.g., a parking lot to a tennis court or play area).

Walking loops are typically 6 ft or wider, and their length varies; they often run around the perimeter of a park or large facilities such as a baseball or sports field. Their surface varies (concrete, asphalt, decomposed granite, dirt or even grass), and some paths include signage to mark distances traveled. Hereafter, we use the term “walking loops” to denote these relatively longer walking paths that are designed for recreational and exercise purposes.

Most parks have short sidewalks, but not all have walking loops which are designed to facilitate people to move continuously along paths without having to stop, thus supporting longer-duration recreational moderate-to-vigorous physical activity (MVPA). Walking loops facilitate people spending time outdoors in natural settings, which is considered healthful (Bowler et al. 2010; Reed et al. 2004), and preliminary evidence suggests that walking loops are associated with increased odds of engaging in MVPA among local residents (Foster et al. 2004; Sugiyama et al. 2015).

Nonetheless, it is possible that walking loops are redundant, because streets and sidewalks also support the same activities. In this study, we used a nationally representative sample of neighborhoods to determine the degree to which park users actually make use of walking loops and whether these enhance or possibly detract from other park uses. New parks are being created and many more are being renovated. If walking loops are associated with higher park use and park-based physical activity, their incorporation into more public parks should be given consideration. Given that nearly half of all Americans fail to adhere to national physical activity guidelines of at least 150 min of MVPA weekly for adults and at least 60 min daily for youth (DHHS 2008), it is critical to identify features that might facilitate more activity (CDC 2015).

## Methods

### Data Sources and Measurement Instrument

The data used in this analysis were fielded in the spring and early summer of 2014 as part of the National Study of Neighborhood Parks, which is described briefly here and available in more detail elsewhere (Cohen et al. 2016). The parent study was determined to be exempt from requirements for human subjects review by the RAND Human Subjects Committee.

We used a two-stage stratified sampling strategy to select a representative sample of neighborhood parks in the U.S. cities with a population of at least 100,000 according to the 2010 Census (U.S. Census Bureau 2010). In the first sampling stage we randomly drew 25 cities from eight strata based on city population (200,000–1,000,000 and 100,000–200,000) and geographic region (West, Northeast, Midwest, and South) and an additional stratum of cities with population > 1 million. All states were in the sampling frame, and by chance all sampled cities were in the 48 continental states. In each of the 25 selected cities we retrieved a list of public parks, either directly from the city’s Department of Recreation and Parks or from their website. We restricted selection to avoid parks in close proximity (< 1 mi from each other) and to ensure that distributions of chosen parks were similar with regard to sizes and local poverty rates for all neighborhood parks within each city. We excluded parks located in a census tract with no or very few residents (e.g., airport, prison, military base, hospital, industrial facility), pocket parks (smaller than 2 or 3 acres), regional parks (larger than 20 or 23 acres in some cities), parks used as school fields during business hours, and parks serving special purposes only (e.g., parkways, boxing gyms). We replaced two parks that police said were unsafe for staff to visit.

We used the System of Observing Play and Recreation in Communities (SOPARC) (McKenzie et al. 2006), which provides aggregated counts of park users by demographics and physical activity levels and characterizes the area contexts in which they are observed. The tool uses momentary time sampling to record observations and has evidence for both reliability (McKenzie et al. 2006) and validity (Evenson et al. 2016; Han et al. 2016). Details about the measurement properties of SOPARC are in the Appendix.

Parks were mapped and divided into target areas, defined as smaller spaces for observation. To help ensure high-quality measurement, all data collectors were centrally trained over a 2-day period. Before collecting SOPARC data, they must have met an accuracy of ≥ 80% for assessing all the key variables (number of park users, sex, age group, and physical activity level). Photos were taken of one target area during each hourly observation, and all data entry and photos were time stamped, allowing us to assess fidelity. Access to these photographs was restricted to study investigators, and these photographs will be destroyed when the study is completed. In addition, unannounced visits were conducted at some parks during data collection to check that the protocols were followed.

Users were enumerated by apparent sex, age group [child (0–12 years), teen (13–19 years), adult (20–59 years), or senior (≥ 60 years)], physical activity, and race/ethnicity (Hispanic, African American, white, and Asian or other). Physical activity categories were defined as sedentary (lying, sitting) or standing, moderate (locomotion from one foot to another at a walking pace), and vigorous (movement greater than a brisk walk). For some analyses we combined moderate and vigorous activity. These observations occurred during 12 hourly observation periods (Cohen et al. 2011) that occurred on Tuesday, Thursday, Saturday, and Sunday at each park at different times of day during daylight hours between April and August 2014. We assessed the use of walking loops by counting people walking past a specific spot during a 10-min period at the end of each of the 12 hourly observations. This is a slightly different protocol than we used in other target areas, in which the entire space was viewed in a single momentary scan. We elected this alternative method because the length of walking loops did not allow for a single momentary scan.

Our main outcome variable, hourly park use, was measured by detailed counts of park users by three-way subcategories defined by demographics (sex and age group) and physical activity levels (sedentary, moderate, and vigorous) during an hourly observation. We also derived a binary outcome of whether or not an entire park was empty (i.e., the total count for the park was zero). To compare levels of physical activity (PA), we converted observed sedentary, moderate, and vigorous intensity into metabolic equivalent (MET)–hours, a measure of energy expenditure, where 1 MET-hr approximates the energy expended for adults during quiet sitting for 1 hr. We assigned 1.5 METs for sedentary, 3.0 METs for moderate PA, and 6.0 METs for vigorous PA (Ainsworth et al. 2000). We summed these to assess overall PA or MVPA in MET-hours for each hourly observation. For example, during an hourly observation, if we observed two sedentary users, two users engaged in moderate PA, and one user in vigorous PA, then the total number of park users is five, the total PA would be 15 MET-hr, and total MVPA would be 12 MET-hr.

We also documented a variety of park conditions, including the accessibility of facilities (yes or no) and the presence of onsite marketing materials, food vendors, apparently homeless individuals, dogs off leash, litter, and graffiti. Finally, to assess whether walking paths were more common in neighborhoods considered to be more walkable, which in itself might explain a greater use of parks with walking loops (Brown et al. 2013; King et al. 2011), we examined the Walk Score® (Walk Score 2015) for all park addresses, a metric that has evidence for validity to indicate neighborhood walkability (Carr et al. 2011; Duncan et al. 2011).

Population density and percent households in poverty in a 1-mi radius from the registered park address were assessed by using U.S. 2010 Census data.

### Statistical Analysis

We first conducted two-sample descriptive statistics to compare park characteristics and park use outcomes between parks with and without walking loops, where we applied two-sample *t*-tests with unpooled variances for continuous variables and *z*-tests for binary variables. We also conducted one-sample descriptive statistics to summarize the use of walking loops alone.

Next, we applied a repeated-measure generalized linear model (negative binomial for most outcomes and logistic for indicators of parks being non-empty) to estimate the relationship between park use outcomes and the indicator for a park having a walking loop, and we adjusted for a list of covariates. These models adjusted for the potential confounders identified in Table 1, including population density and percentage of households in poverty within a 1-mi radius of the park; number of accessible target areas; number of target areas having supervised activities; number of types of facilities; maximum, minimum, and mean temperatures collected from the National Oceanic and Atmospheric Administration (NOAA) on the days the parks were observed; presence of (*a*) onsite marketing materials such as banners, signage, and posters, (*b*) moderate or more litter, (*c*) homeless people, (*d*) food vendors in or around the park, (*e*) dogs off leash, and (*f*) moderate or more graffiti; as well as fixed effects for cities, days of a week, and hours of a day. These model estimates were multiplicative effects (percent changes for counts, or odds ratios for binary outcomes).

**Table 1:** Comparison of characteristics between parks with and without walking loops (mean ± SD or percent).

Park characteristic^*a*^	With walking loop (*n* = 50 parks)	Without walking loop (*n* = 124 parks)^*b*^
Size (acres)	9.4 ± 5.3	8.6 ± 5.6
Population within 1-mi radius (*n *× 1,000)	28.8 ± 38.4	22.3 ± 31.1
Percent poverty in 1-mi radius	19.0	20.1
Accessible target areas (*n*)	21.2 ± 15.6	20.1 ± 13.1
Target areas having supervised activities (*n*)	0.4 ± 1.5	0.5 ± 1.2
Types of facilities (*n*)	10.4 ± 10.4	9.3 ± 7.4
Onsite marketing materials such as banners, signage, posters (% parks)	22.0	30.0*
Moderate or more litter in parks observed at least once (% parks)	38.0	37.8
Homeless people observed at least once (% parks)	28.0	27.4
Food vendors observed at least once (% parks)	26.0	27.4
Dogs off leash observed at least once (% parks)	66.0	57.3*
Moderate or more graffiti observed at least once (% parks)	14.0	7.6**
Maximum temperature (°F)	78.4 ± 10.8	78.9 ± 10.1
Minimum temperature (°F)	55.7 ± 10.5	56.8 ± 10.4
Mean temperature (°F)	67.1 ± 10.0	67.9 ± 9.4
Walk Score^®^	48.1 ± 29.2	46.4 ± 25.5
^***a***^Acres, population within 1-mi radius, and poverty rate are time invariant. Numbers of target areas accessible and having supervised activities were based on hourly observations. All other characteristics were based on daily observations. ^***b***^Significant differences between the two sets of parks are presented as **p < 0.01, *p < 0.05.		

## Results

### Descriptive Statistics

Table 1 presents the comparisons of park characteristics between parks with and without walking loops. Of the 174 parks, 50 had a walking loop; 48 of these were circular and 2 were linear, including 1 that was part of a larger trail system that traversed the park. Parks with and without walking loops were similar in size, neighborhood population density, percentage of households in poverty; types of park facilities; supervised and accessible target areas; presence of food vendors, litter, and apparently homeless individuals; maximum, minimum, and average temperature; and Walk Score®. However, parks with walking loops were less likely to have marketing materials and more likely to have moderate or more graffiti and dogs off leash.

Table 2 presents the hourly use of walking loops. Walking loops themselves were vacant during 35% of the hourly observations, compared with an average vacancy rate of 75% across all target areas. Average hourly use for a walking loop was 3.8 persons/hr, with males using them more often than females (2.1 vs. 1.6/hr, *p* < 0.001). More adults used the walking loops than youths or seniors (*p* < 0.001). Moderate PA dominated (76%) the total use on walking loops, whereas 18% were engaged in vigorous activity and 5% were sedentary (e.g., pushed in stroller or wheelchair).

**Table 2:** The use of walking loops among study parks with this feature (*n* = 50 parks).

Park use measurement	Sample mean (%)
Loops empty (% of observations)	35.1
Average use per hour	3.8 persons/hr
Average use per week	291 persons hr/week
Average use by sex
Females	1.6/hr (43)
Males	2.1/hr (57)
Average use by age group
Children	1.0/hr (27)
Teens	0.4/hr (11)
Adults	2.0/hr (54)
Seniors	0.3/hr (8)
Apparent race/ethnicity of users
Latino	1.1/hr (29)
African American	0.9/hr (24)
White	1.5/hr (39)
Asian and other	0.3/hr (8)
Observed activity levels of users
Sedentary	0.2/hr (5)
Moderate	2.9/hr (76)
Vigorous	0.7/hr (18)

Table 3 shows the unadjusted comparison between parks with and without walking loops. Those with walking loops were more likely to be occupied (86.8% vs. 70.8% among all hourly observations, *p* < 0.001), and on average parks with walking loops had approximately eight more users per hour (42.4 vs. 34.4 during an hourly observation, *p* < 0.10). Given that the average count of users on walking loops was 3.8 persons/hr (Table 2), we estimated that 4.2 additional users/hr were counted in other park areas.

**Table 3:** Unadjusted two-sample comparisons of number of park users and their level of physical activity observed in parks with and without walking loops (mean ± SD).

Outcomes	Sex	Age group	Parks with a walking loop (*n* = 50)	Parks without a walking loop (*n* = 124)^*a*^
Park non-empty (% hourly observations)			86.8	70.8***
Park users (*n *users/hr)	Female	Children	5.6 ± 13.2	4.3 ± 11.9*
		Teenagers	2.9 ± 10.7	1.9 ± 7.4*
		Adults	8.8 ± 20.8	7.2 ± 20.5
		Seniors	0.9 ± 3.1	0.6 ± 2.4*
	Male	Children	7.8 ± 19.7	6.9 ± 19.0
		Teenagers	4.5 ± 13.0	3.4 ± 10.6^#^
		Adults	10.5 ± 21.8	9.3 ± 22.6
		Seniors	1.3 ± 3.1	0.7 ± 2.6***
	Total		42.4 ± 92.5	34.4 ± 85.4^#^
Moderate to vigorous physical activity (in MET-hr)	Female	Children	9.9 ± 23.7	6.9 ± 19.4**
		Teenagers	5.0 ± 25.2	2.5 ± 10.0*
		Adults	9.3 ± 26.9	5.2 ± 14.3***
		Seniors	1.0 ± 3.7	0.4 ± 1.6***
	Male	Children	14.5 ± 35.6	11.8 ± 30.9^#^
		Teenagers	7.9 ± 27.9	6.2 ± 20.9
		Adults	14.4 ± 32.5	10.0 ± 24.0**
		Seniors	1.4 ± 3.6	0.6 ± 2.6***
	Total		63.4 ± 145.8	43.5 ± 98.8**
^***a***^Significant differences between the two sets of parks are presented as ***p < 0.001, **p < 0.01, *p < 0.05, ^#^*p *< 0.10.				

The difference in MVPA between parks with and without walking loops is even greater: the total hourly MVPA was 63.4 and 43.5 MET-hr for parks with and without trails (*p* < 0.01), respectively (Table 3). Parks with walking loops had more observed users accruing more MET-hours in MVPA in most age and sex groups.

### Model Results

With adjustment, we found that parks with walking loops had 80% more users (*p* < 0.001, 95% confidence interval (CI): 42, 139%) and they accrued 90% more MET-hours (*p* < 0.001, 95% CI: 49, 145%) than parks without walking loops (Table 4). In addition, the odds of the park being occupied were 2.6 times higher when a walking loop was present (*p* < 0.001, 95% CI: 1.6, 4.2). The largest impact of walking loops was for seniors (Table 4). Considering entire parks, female and male seniors engaged in 3.6 and 3.9 times more MVPA, respectively, than their senior peers in parks without walking loops.

**Table 4:** Adjusted estimates for impact of walking loops on park use and physical activity.*^a^*

Effects	Sex	Age group	Estimate (SE)^*b*^
Ratios in number of park users per hour between parks with and without walking loops	Female	Children	1.8 (0.3)**
		Teenagers	1.6 (0.3)*
		Adults	1.9 (0.3)***
		Seniors	1.9 (0.3)***
	Male	Children	2.0 (0.4)***
		Teenagers	1.1 (0.2)
		Adults	1.5 (0.2)**
		Seniors	2.7 (0.4)
	Total		1.8 (0.2)***
Odds ratio of park being non-empty during observations			2.6 (0.6)***
Ratios in METs spent in MVPA per hour between parks with and without walking loops	Female	Children	1.8 (0.4)**
		Teenagers	1.8 (0.4)*
		Adults	2.5 (0.4)***
		Seniors	3.6 (1.0)***
	Male	Children	1.9 (0.4)**
		Teenagers	1.2 (0.3)
		Adults	1.8 (0.2)***
		Seniors	3.9 (1.0)***
	Total		1.9 (0.2)***
^***a***^Models adjusted for covariates in Table 1 as well as fixed effects for cities, day of a week, and hours of a day. Correlations among repeated measures in a park were handled by generalized estimating equations. ^***b***^Statistical significance: ***p < 0.001, **p < 0.01, *p < 0.05.			

## Discussion

These findings indicate that at the national level, parks with walking loops had more visitors than parks without them. Although the evidence is based on a cross-sectional study using observations and causality cannot be established, having a walking loop might boost overall park use for several reasons. For example, the walking loops in parks may be in better condition than city streets and sidewalks (e.g., sidewalks frequently have uneven, cracking surfaces; often have driveway ramps, partly blocked; are noisy, and are unprotected from traffic). Walking loop users may also be less worried about potential collisions. These may be an important reason why parks with walking loops attract relatively more seniors, who may lack confidence about their ability to navigate streets and sidewalks with defects and safety hazards.

Given no difference in the walkability of neighborhoods as measured by Walk Score® for parks with and without walking loops, walking loops are likely not being used to compensate for streets and sidewalks that are not pedestrian friendly. We conjecture that walking loops in a park might provide both physical and psychological advantages, in that they could attract a regular community of users who get to know each other and provide social support. Additionally, a park with diverse facilities can cater to many different users, and walking loops may enhance the overall attractiveness of a park and increase its use.

The additional persons observed in parks with walking loops were not limited to using the walking loops. Parks with walking loops typically have multiple other facilities, and it is these complementary, accompanying facilities that accounted for approximately half the additional users in the park (4.5 of the additional 8 users/hr), rather than the loop itself.

However, walking loops could attract adult caregivers to stay and use them, while their children are actively engaged in playgrounds and sport fields.

Seniors did use parks with walking loops more than those without them, but their overall representation in parks is still exceedingly low. Seniors comprise about 20% of the general U.S. population, but only about 8% of walking loop users and 4% of the overall park users in this representative sample of U.S. neighborhood parks were seniors. However, seniors suffer from higher rates of disability and may have more ambulatory limitations than younger individuals (Ferrucci et al. 2016), which may partly explain their lower use of parks. This would also suggest that walking loops should be able to accommodate assisted ambulation devices such as walkers.

Increasing access to places where people can engage in PA has also been found to be an effective intervention (Kahn et al. 2002; Krieger et al. 2009). Walking loops fulfill that need by making parks more pedestrian friendly. A study by Powell et al. (2003) showed that having access to public parks and walking or jogging trails was associated with a higher percentage of people meeting national PA guidelines compared with those who lacked access to places to walk. Our findings provide additional evidence that access to walking loops supports more MVPA.

### Limitations

There are a couple of caveats to this study. First, our assessment of walking loops used a slightly different protocol than those used for other target areas. Depending on the length of the walking loop, our procedure might underestimate use if people were moving slowly or overestimate use if people were moving faster. For shorter trails, overcounting was likely a problem. Second, because walking loops were not randomized to study parks, we cannot establish a causal relationship between their existence and the outcomes studied. Instead, we can only confirm the association between the presence of walking loops and a greater number of park users. Third, although the measures of the total number of park users are robust, estimates of MVPA have somewhat lower reliability (Cohen et al. 2011). Other unmeasured factors such as a favorable location or even the presence of unique landscaping and vegetation may be the real cause leading to the significant association between walking loops and an outcome. Reverse causality is also possible: Walking loops could have been built because more people were already using these parks and there were demands for additional facilities. Thus, walking loops could have been a consequence of, rather than a cause for, the higher rates of park use.

Future studies with a longitudinal design may be able to confirm the benefits that walking loops in parks offer. For example, an interrupted time series analysis or a difference-in-differences analysis could be applied to examine the causal effect of newly built walking loops in parks compared to similar parks without walking loops.

## Conclusion

In contrast to other park facilities that support PA (e.g., gymnasia, swimming pools, skate parks), walking loops may be relatively inexpensive additions that could be placed around the perimeter of many parks (Brownson et al. 2000; Hunter et al. 2013). They are particularly beneficial to seniors, who may prefer them because of the relative increased safety of being able to walk on a smooth, uninterrupted path (Rosenberg et al. 2013) that is away from motor vehicle traffic (Gallagher et al. 2010). One research synthesis found that safety was more important than park proximity in fostering walking among seniors (Yen et al. 2014). Given the decline in PA with age, walking loops may be an important, feasible, and affordable remedy to widespread lack of PA that occurs not only among seniors (Michael et al. 2006), but also among the population in general.

Appendix **Reliability and Validity of Measures**

The reliability of SOPARC was established comparing the responses of independent raters (Cohen et al. 2011; Han et al. 2016). Inter-rater agreements for park user characteristics between two proficient observers were high—averaging 94%, with a range of 85–99%. When only the instances when the target areas were not empty were examined, average agreement on specific park user characteristics was 87% for the total number of individuals, 82% for race/ethnicity, 82% for age group, and 80% for physical activity level—a high level of concordance. Agreement between two proficient observers in assessing total METs and METs in all age and sex categories were also high (between 82% and 97%) except for male seniors (between 64% and 86%). To determine the minimum hourly observations needed to estimate weekly park use, we measured park use for 14 hr per day for 14 days in 10 neighborhood parks in five cities. For using 12 hourly observations to estimate the weekly use of the park, we calculated the intraclass correlation coefficient (ICC) as 0.86 for the number of users, 0.86 for sedentary users, and 0.82 for moderate and vigorous users (Cohen et al. 2011).

An inter-instrument validity check compared pictures of target areas with observed counts in the field, where the pictures were taken simultaneously with the observations. Analysis was limited to total number of persons and total METs (assigned to correspond to sedentary, moderate, or vigorous intensity) of an area, because the pictures did not have sufficient details to discern the age and sex of every person. The correlation between the picture-based measurements and field measurements by the 12-button counters was 0.94 for the total number of persons and 0.80 for total METs. The ICC was 0.92 for the total number of persons and 0.79 for total METs (Han et al. 2016).

Other researchers have also found the SOPARC method assessment reliable (Bocarro et al. 2009; Evenson et al. 2016).
